# Application of Metal-Based Nanozymes in Inflammatory Disease: A Review

**DOI:** 10.3389/fbioe.2022.920213

**Published:** 2022-06-16

**Authors:** Ruifeng Li, Xinyue Hou, Lingrui Li, Jiancheng Guo, Wei Jiang, Wenjun Shang

**Affiliations:** ^1^ Application Center for Precision Medicine, Department of Molecular Pathology, The Second Affiliated Hospital of Zhengzhou University, Academy of Medical Sciences, Zhengzhou University, Zhengzhou, China; ^2^ Department of Kidney Transplantation, The First Affiliated Hospital of Zhengzhou University, Academy of Medical Sciences, Zhengzhou University, Zhengzhou, China

**Keywords:** reactive oxygen species, inflammatory diseases, antioxidant, metal-based nanozyme, enzyme mimic activity

## Abstract

Reactive oxygen species (ROS) are metabolites of normal cells in organisms, and normal levels of ROS in cells are essential for maintaining cell signaling and other intracellular functions. However, excessive inflammation and ischemia-reperfusion can cause an imbalance of tissue redox balance, and oxidative stress occurs in a tissue, resulting in a large amount of ROS, causing direct tissue damage. The production of many diseases is associated with excess ROS, such as stroke, sepsis, Alzheimer’s disease, and Parkinson’s disease. With the rapid development of nanomedicine, nanomaterials have been widely used to effectively treat various inflammatory diseases due to their superior physical and chemical properties. In this review, we summarize the application of some representative metal-based nanozymes in inflammatory diseases. In addition, we discuss the application of various novel nanomaterials for different therapies and the prospects of using nanoparticles (NPs) as biomedical materials.

## Graphical Abstract

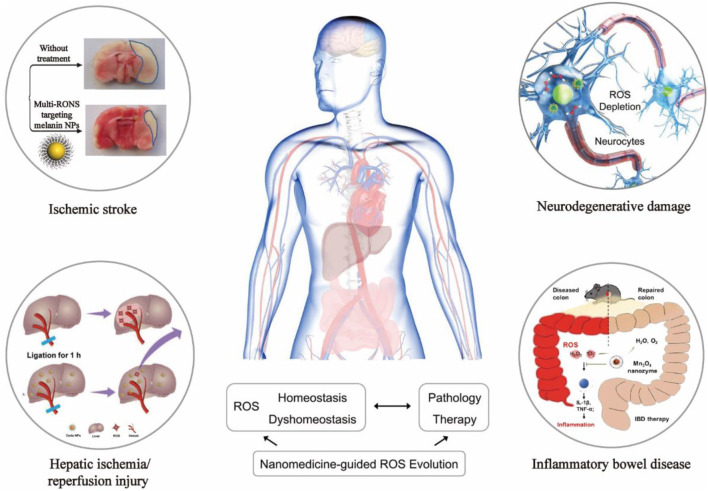

## 1 Introduction

**FIGURE 1:**
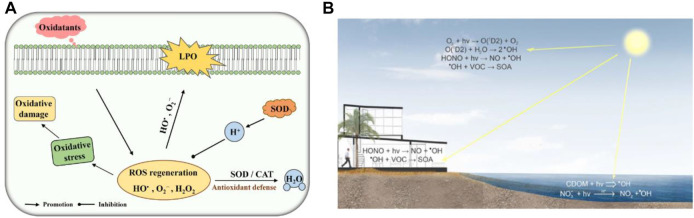
**(A)** Schematic diagram of SOD and CAT toxicity against ROS. LPO, lipid peroxidation. Adapted with permission from ref.19. Copyright (2020) Frontiers in Microbiology. **(B)** ROS Material Chemistry. Adapted with permission from ref.11. Copyright (2016) American Chemical Society.

The oxygen-containing mono-electron by-products produced by cells in the process of respiration and organism metabolism are called reactive oxygen species (ROS) (Panth et al., 2016). ROS are composed of superoxide-free radicals, hydroxyl-free radicals, peroxide-free radicals, hydrogen peroxide, hypochlorous acid, and ozone (Panth et al., 2016) (Figure 1). ROS production and scavenging maintain a dynamic balance. As a result, the cell’s antioxidant system is disrupted, prompting the cell to produce excessive ROS, which destroys the redox state and causes oxidative stress. The oxidative stress can cause severe damage to cells, leading to cell structure damaged by an injury to the cell components, including the cell membrane and nucleus (Floyd and Carney, 1992; Cabiscol et al., 2000; Leutner et al., 2001). This damage to the cell structure leads to damage to cell function that results in a series of serious diseases, for instance, Parkinson’s disease, heart/kidney ischemia-reperfusion injury, diabetes, inflammation, cardiovascular disease, and cancer (Barnham et al., 2004; Giordano, 2005; Rolo and Palmeira, 2006; Jaeschke, 2011; Wang and Choudhary, 2011).

ROS are generated by both intracellular and extracellular metabolic pathways (Gligorovski et al., 2015; Nosaka and Nosaka, 2016). Substances that eliminate, inhibit, and prevent ROS from reacting with cells are called antioxidants (Flora, 2007; D'Autréaux and Toledano, 2007; Lambeth, 2004). Antioxidants are used to remove excess reactive oxygen species produced in living organisms. There are a few free radical scavengers in organisms’ antioxidant systems: endogenous free radical scavengers such as superoxide dismutase (SOD) and vitamin E, exogenous free radical scavengers such as polyphenols, and Chinese herbal medicine that has antioxidation action similar to glossy *Ganoderma* and *Salvia miltiorrhiza*. Furthermore, the natural antioxidant enzyme system catalyzes free reactions to produce harmless products in removing ROS and reducing the damage caused by ROS. Among them, the leading natural antioxidant enzymes include superoxide dismutase (SOD), catalase (CAT), peroxidase (POD), and glutathione peroxidase (GPx) (Valko et al., 2006; Tidwell, 2013; Hamada et al., 2014; Kong et al., 2020; Hou et al., 2021). Although traditional natural enzymatic antioxidants are widely used, they are easily oxidized and have low bioavailability, poor modification, and stability. In addition, they are challenging to target scavenging oxygen free radicals, challenging to cross the blood–brain barrier, and easy to be neutralized by cell culture medium. To make up for the deficiency of traditional natural enzymes, several studies find the substitutes or mimic enzymes that can compensate for the shortcomings of natural enzymes and develop new antioxidants to make them better applied in production and life (Wu et al., 2019).

**FIGURE 2:**
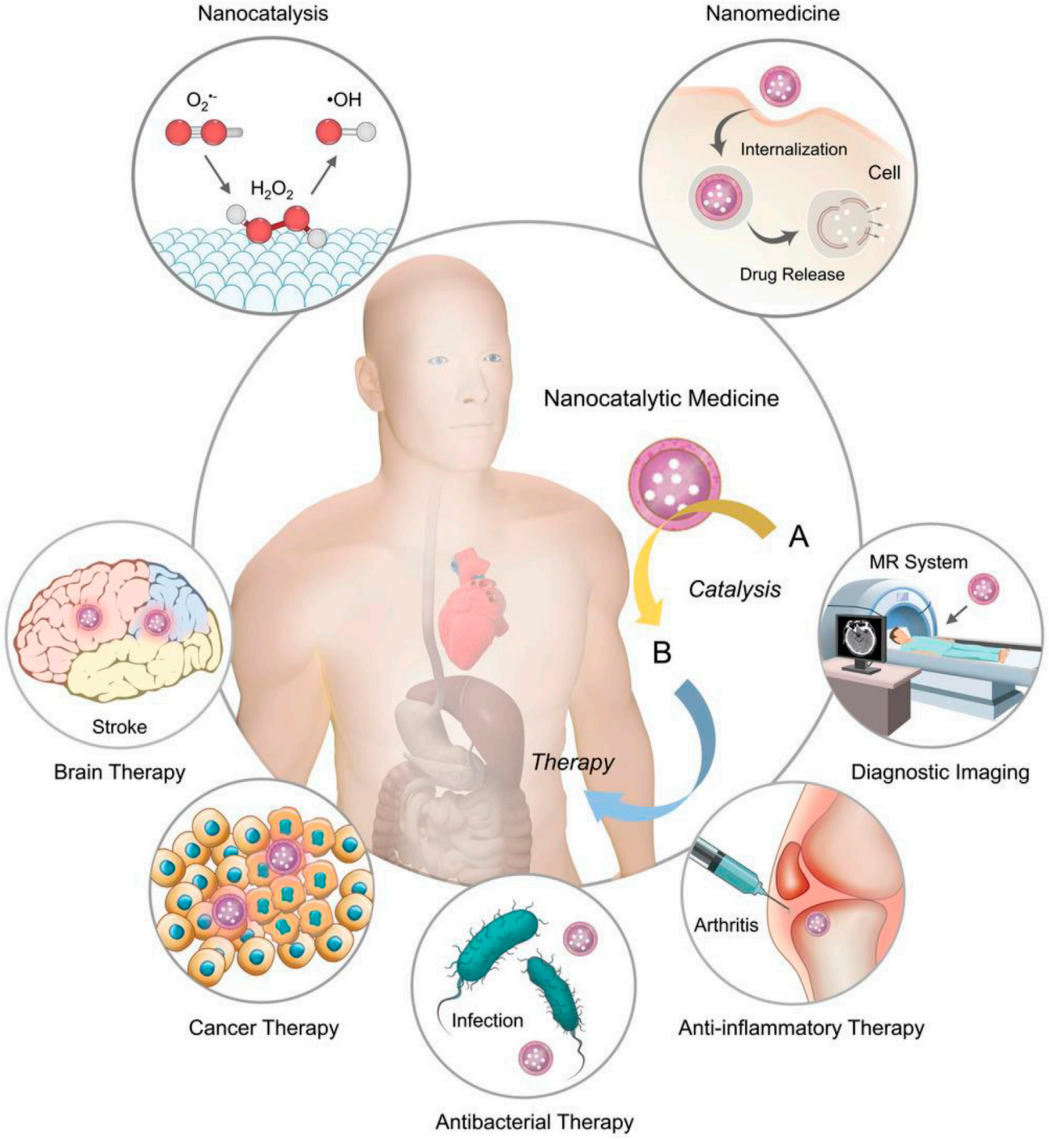
Combining nanocatalysis with clinical medicine provides an advanced chemical modulation method for clinical treatment. MR, magnetic resonance. Adapted with permission from ref.17. Copyright (2019) John Wiley and Sons.

With the rapid development of nanotechnology, nanomaterials have been widely used in biomedical, optics, catalysis, and other fields because of their excellent physical and chemical properties, their ability to penetrate cell membranes, high activity, and low production cost (Yang et al., 2019; Liu et al., 2021; Yi et al., 2022). Because researchers found that these nanoparticles have the inherent ability to mimic the catalytic activity of certain biological enzymes, they are called nanomimicase enzymes, or nanozymes in short. In 2007, Xi Yunyan et al. found that iron tetroxide nanoparticles show natural HRP activity, which can catalyze the reaction between a substrate and hydrogen peroxide. Thus, they demonstrated that the nanomaterials themselves could simulate the functional activity of some biological enzymes (Gao et al., 2007). In previous studies, nanomaterials, such as fullerenes, gold nanoparticles, and ferromagnetic nanoparticles, also have been found to have the activity of some natural enzymes. These nanomaterials with the activity of natural enzymes are called nanozyme (Dugan et al., 1996; Comotti et al., 2004; Manea et al., 2004; Li et al., 2015), and nanozyme antioxidants, as nanozyme preparation, make up for the deficiency of traditional natural enzymes. In addition, nanozyme antioxidants have the advantages of low production cost, high modification degree and surface activity, targeted enrichment in specific tissues, high biocompatibility, and scale production. Thus, these advantages make the nanozyme antioxidants widely used in cancer treatment, biological science, drug carrier, biological antioxidant, and other fields. Nanoparticles could be a potential efficient therapeutic option for clinical treatment because they alter the biological distribution of antioxidants and have the inherent ability to remove electrons. According to the different catalytic substrates, the existing nanozymes can be divided into mimic peroxidase enzymes, mimic oxidase enzymes, catalase mimic enzymes, and superoxide dismutase mimic enzymes. Among them, peroxidase can catalyze the oxidation of hydrogen peroxide oxidation substrate; the oxide mimic enzyme catalyzes the oxidation of oxygen to the substrate; the CAT enzyme can catalyze the hydrogen peroxide decomposition reaction; and SOD enzymes can catalyze the superoxide anion disproportionation to produce hydrogen peroxide and oxygen. Therefore, these NPs could be applied to disease diagnosis, treatment, and biomedicine (Duan et al., 2015; Wang et al., 2016a; Cervadoro et al., 2018) (Figure 2).

According to recent studies, three kinds of nanozymes are commonly used: Prussian blue, nanocerium dioxide particles, and manganese-based analyses. In 1996, the Okuda team found that fullerenes could eliminate the superoxide radicals (Okuda et al., 1996); the Seal team (2005) showed that nanocerium dioxide (CeO_2_) could prevent cell damage by radiation and attributed to the scavenging of free radicals (Okuda et al., 1996). Additionally, Gu et al. (2016) revealed that Prussian blue nanoparticles (PBNPs) have properties of catalase and superoxide dismutase (Watanabe et al., 2009). Qu Xiaogang and Ren Jinsong (2016) constructed a Donanamil complex system, which effectively eliminates overexpressed ROS in cells and prevents cells from oxidative stress damage (Huang et al., 2016). This article reviewed the types of nanoparticles with antioxidant capacity, the mechanism of action of nanozymes, and their clinical applications. This article mainly introduces the research progress of manganese NPs, ceria NPs, Prussian blue NPs, and other NPs in inflammation, Parkinson’s disease, brain/liver ischemia/reperfusion, and other diseases.

### 1.1 Manganese-Based NPs

**FIGURE 3:**
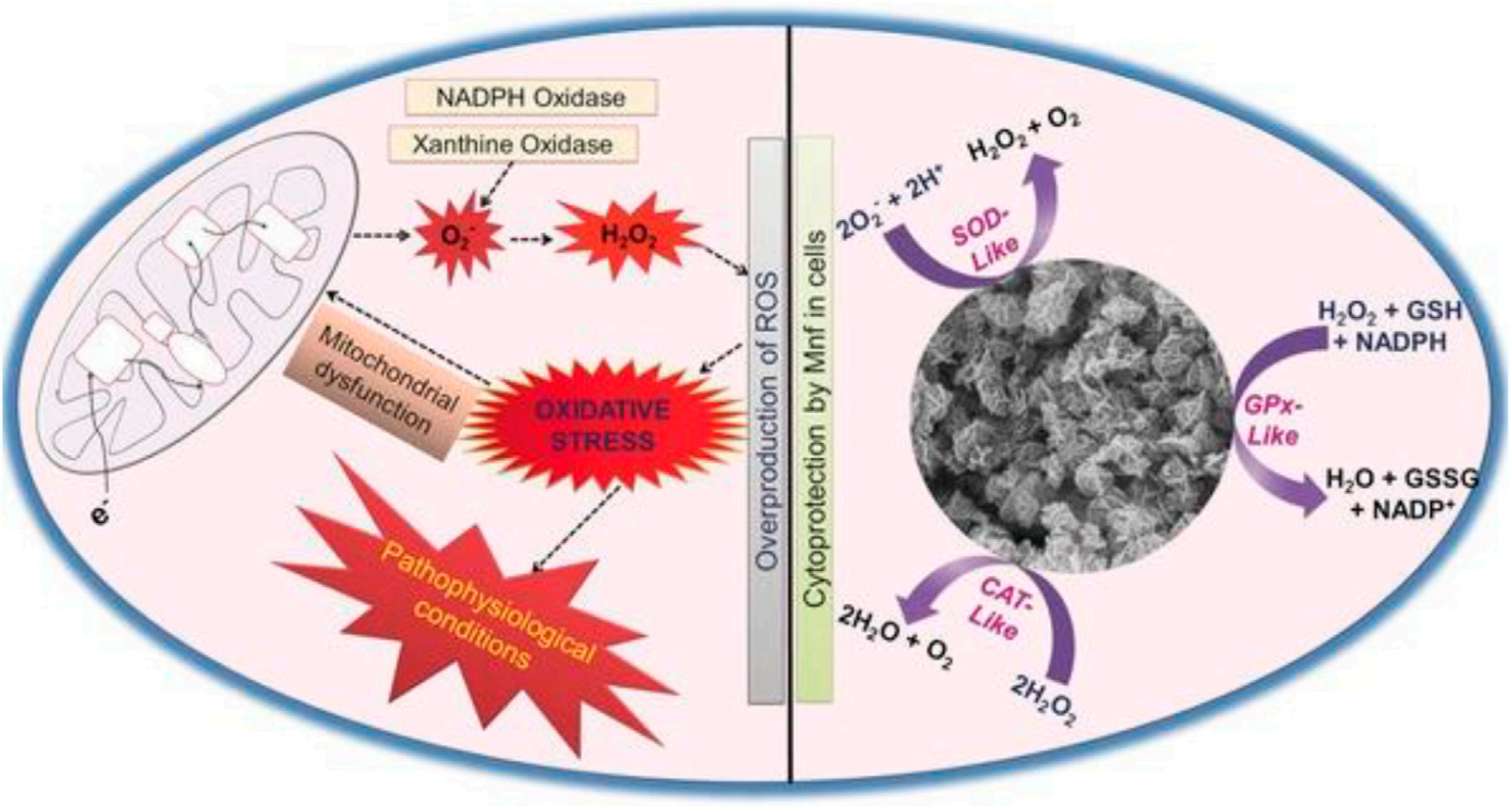
Mn_3_O_4_ nanozyme (Mnf) modulates intracellular redox balance by mimicking the activities of catalase (CAT), glutathione peroxidase (GPx), and superoxide dismutase (SOD). Adapted with permission from ref.33. Copyright (2017) John Wiley and Sons.

Manganese-based nanomaterials are nanomaterials with manganese as the active center and natural enzyme activity (Huang et al., 2016). In addition to playing an essential role in photosynthesis, divalent manganese plays a crucial role in hydrolysis and phosphotransferase. The more expensive manganese is the redox center of ribonucleotide reductase, catalase, peroxidase, and SOD in the mitochondria (Li et al., 2017; Wan et al., 2012). Manganese phosphate (Mn_3_(PO_4_)_2_) is the first reported manganese nanoscale with superoxide dismutase activity (Miriyala et al., 2012). In 2014 (Figure 3), Wu et al. used bovine serum protein to wrap manganese dioxide (BSA-MnO_2_), which can regulate the anaerobic status of the tumor microenvironment and has a specific effect on tumor treatment (Prasad et al., 2014). In 2016, researchers found that octahedral manganese oxide (MnO) nanomaterials have the properties of superoxide dismutase, and the relaxation time of MnO increases when it is in contact with superoxide radicals (Prasad et al., 2014). In addition, the researchers found that the dendritic structure of Mn_3_O_4_ nanoparticles had a larger pore size and activities of superoxide dismutase and catalase (Yan et al., 2013).

Furthermore, the elimination rate of Mn_3_O_4_ on the superoxide anion can reach 50–60% (Singh et al., 2017). These advantages make up for the defects of natural enzyme antioxidants, so the researchers have researched the application of manganese-based nanozymes in treating clinical diseases and provided corresponding theoretical support for the application of manganese-based nanozymes in clinical treatment. Based on this, this article reviews the recent progress in synthesizing Mn_3_O_4_ nanoparticles by relevant teams in treating Parkinson’s disease, inflammation, and other diseases.

Parkinson’s disease (PD) is a neurodegenerative disease characterized by the substantia nigra striatum pathway degeneration, closely related to oxidative stress (Barnham et al., 2004). In the brain, dysregulation of redox balance can lead to oxidative stress in neuronal cells, resulting in neuronal loss (Yan et al., 2013; Li et al., 2020). Currently, there is no effective clinical treatment for PD. Moreover, the treatment for the disease is also the focus of clinicians and scientists. A few nanomaterials have been reported to mimic the activity of heme peroxidase, CAT, oxidases, or SOD enzymes (Gao et al., 2007; Asati et al., 2009; Wei and Wang, 2013; Dong et al., 2014). In addition, it has been reported that under pathophysiological conditions, the proper control of H_2_O_2_ levels through CAT–GPx cooperativity is essential (Baud et al., 2004). Given the *in vitro* antioxidant enzyme activity of the nanomaterials, Namrata Singh et al. combined the characteristics of enzyme-active nanomaterials to synthesize flower-shaped (Mnf) Mn_3_O_4_ nanoparticles. They established the cell model of Parkinson’s disease in *in vitro* experiments, providing a clinical reference for treating Parkinson’s and other neurological diseases. The activities of CAT, SOD, and Gpx of Mnf nanoparticles were verified by the comprehensive evaluation and characterization of Mnf nanoparticles’ antioxidant capacity and different morphology Mn_3_O_4_ nanoparticles (Singh et al., 2017). The phase and morphology of Mnf did not change during the reaction, which proved that the high activity of Mnf was closely related to its morphology and multi-pore size. Human neuroblastoma-derived cell line SHSY-5Y was selected in the cell experiment, and the PD cell model was established by using the neurotoxin MPP^+^ targeting dopamine neurons (Singh et al., 2017). MTT verified that Mnf nanoparticles were nontoxic, and the results of the DCFDA-H2 probe experiment showed that the loss of neuronal cell processes induced by MPP+ was repaired, confirming the protective effect of nanoparticles on cells. More importantly, the research results of Namrata Singh et al. proved that Mnf has better scavenging capacity of reactive oxygen species than traditional natural enzymes, which provides the effective reference data and theoretical guidance for the clinical application of Mnf in the treatment of neurodegenerative diseases caused by oxidative stress.

Inflammation has been demonstrated to cause various diseases, such as rheumatoid arthritis (Choy and Panayi, 2001), cardiovascular diseases (Taube et al., 2012), and even cancer (Colotta et al., 2009). The inflammatory response in the body is also closely related to reactive oxygen species. The production of free radicals in inflammatory sites is one of the pathogenesis of diseases. Mn_3_O_4_ nanoparticles (NPS) have a variety of enzyme activities to simulate the removal of oxygen free radicals, hydrogen peroxide, and hydroxyl radicals (Gorecki et al., 1991; Miriyala et al., 2012). Jia Yao et al. synthesized Mn_3_O_4_ NPs by the hydrothermal method and found that Mn_3_O_4_ NPs are more stable and have higher activity than natural enzymes, showing a good ROS scavenging effect *in vitro* (Yoshimura et al., 2021). These findings provide a basis for clinical studies on ROS-mediated inflammatory use of drugs. The DLS and zeta potential results proved that Mn_3_O_4_ NPs had good long-term storage stability. Also, the high crystallinity was confirmed by TEM detection (Yao et al., 2018; Yoshimura et al., 2021). The CO_2_-specific probe hydroethidine (HE) was used to characterize the CO_2_ scavenging capacity of Mn_3_O_4_ NPs. The experimental results showed that Mn_3_O_4_ NPs had a better SOD-like activity. Terephthalic acid (TA) reacted with hydrogen peroxide to prove that Mn_3_O_4_ NPs had a Catalin-like activity. The absorption spectrum and EPR spectrum were used to detect the hydroxyl group level in the presence of Mn_3_O_4_ NPs. In *in vitro* experiments, PMA was applied to the ears of mice, and the local inflammatory response was typical in the treated area. The fluorescence intensity of the DCFDA-H2 probe before and after comparison proved the scavenging ability of Mn_3_O_4_ NP reactive oxygen species, and HE staining showed that PMA induced lymphocytosis in the ear of mice, and the ear treated with Mn_3_O_4_ NPs could significantly alleviate the inflammatory symptoms. Jia Yao et al. not only demonstrated the scavenging activity of Mn_3_O_4_ analyses but also provided a promising therapeutic strategy for redox using the redox active analyses.

Inflammatory bowel disease (IBD) is a nonspecific chronic inflammatory disease of the intestinal tract, mainly including ulcerative colitis (UC) and Crohn’s disease (Sheng et al., 2019; Chen and Shen, 2021). The pathogenesis of IBD is very complex and is generally believed to be related to immune, environmental factors, and genetic and genetic defects or changes (Podolsky, 2002). There are few effective treatments for IBD. Oxidative stress is a fundamental cause of IBD and plays an essential role in some of the characteristic signs and symptoms of IBD, such as abdominal pain, diarrhea, and big toxic colon (Zhao et al., 2019). Excessive ROS production leads to oxidative damage to the DNA, proteins, and lipids, which may promote the initiation and development of IBD (Mittler, 2017). Therefore, targeting inflammatory sites and scavenging reactive oxygen species may be effective strategies to reduce IBD. Natural enzymes in organisms, such as superoxide dismutase and catalase, can precisely remove O_2_^−^ and H_2_O_2_, respectively, to protect the body from ROS damage. However, ROS in the focal area of IBD is often excessive, and natural enzymes in the organism are often tricky to control ROS in the normal physiological range, leading to the difficulty in clearing inflammation. At the same time, due to the high specificity of natural enzymes, a natural enzyme usually can selectively catalyze only one substrate, and it is challenging to remove multiple ROS simultaneously. In order to make Mn_3_O_4_@OLA@DSPE-PEG-COOH (hereinafter referred to as DPOMn_3_O_4_ nanoenzymes) which is stable in aqueous solution, Jia Yao et al. modified the surface of Mn_3_O_4_@OLA by using clear, good biocompatibility, and structure of small molecule lecithin derivative two stearic acyl phosphatidyl ethanolamine carboxy end group-polyethylene glycol (DSPEPEG-COOH) group. They confirmed the ability of the DPO-Mn_3_O_4_ nanoenzyme to scavenge superoxide anions *in vitro*. Subsequently, inflammatory bowel disease induced by dextran sulfate sodium (DSS) was established in mice (Mittler, 2017). Through enema administration of the DPO-Mn_3_O_4_ nanoenzyme, the therapeutic effect of DPO-Mn_3_O_4_ on the inflammatory bowel disease in mice was verified by the direct observation of the length and swelling degree of the colon of mice and the observation of colon tissue sections. Therefore, Yao Jia et al. demonstrated that Mn_3_O_4_ nanoenzyme could treat local *in vivo* inflammation by scavenging ROS.

### 1.2 Cerium-Based NPs

Cerium oxide (CeO_2_) nanoparticles, also called nanoparticles of cerium oxide nanoparticles (CNPs), are well-known catalysts that show significant pharmacological potential due to their antioxidant properties (Ferreira et al., 2018). Cerium oxide itself has oxidation, as a combustion catalyst or catalyst carrier; which is widely used to remove industrial harmful gases or formaldehyde in the room, also is favored in the chemical industry. Compared with the oxidability of ceria, nanoceria has oxidability and reducibility. Oxygen in the nanometer cerium oxide lattice quickly falls off, resulting in oxygen hole defects. For the charge balance of the crystal, a small amount of Ce^4+^ is converted to Ce^3+^. Ce^3+^ is reductive and will be oxidized to Ce^4+^ in the oxidation condition.

Consequently, Ce^3+^ is easy to be oxidized, and Ce^3+^ itself is reductive (Celardo et al., 2011). Therefore, CNPs scavenge free radicals by reversibly binding oxygen through moving between Ce^3+^(reduction) and Ce^4+^(oxidation) forms on the particle surface. This ability is comparable to that of biological antioxidants. Because of this, CNPs are believed to exhibit the activity similar to dismutase and catalase, protecting cells from superoxide ions but hydrogen peroxide, two significant reactive oxygen species (Korsvik et al., 2007; Singh et al., 2011). Ceria nanoparticles are widely used in biomedicine for treating antioxidant-related diseases. Confirmed by the electron spin resonance, cerium oxide has the nature of the scavenging free radicals (Colon et al., 2009). In 2009, the United States, led by J Colon, consisting of the chemical, medical research team, provided in the United States “nano-drug” academic journals, published an article “using cerium oxide nanoparticles that protect normal cells from pneumonia caused by radioactive,” which created new in the field of nanometer cerium oxide in biomedical applications (Colon et al., 2009; Colon et al., 2010). With the increasing popularity of cerium oxide nanoparticles in clinical use, the research team carried out relevant research on the application of cerium oxide in the treatment of liver ischemia/reperfusion. Moreover, the treatment of ischemic stroke also made significant progress. Consequently, this article reviewed the research results of relevant teams.

**FIGURE 4:**
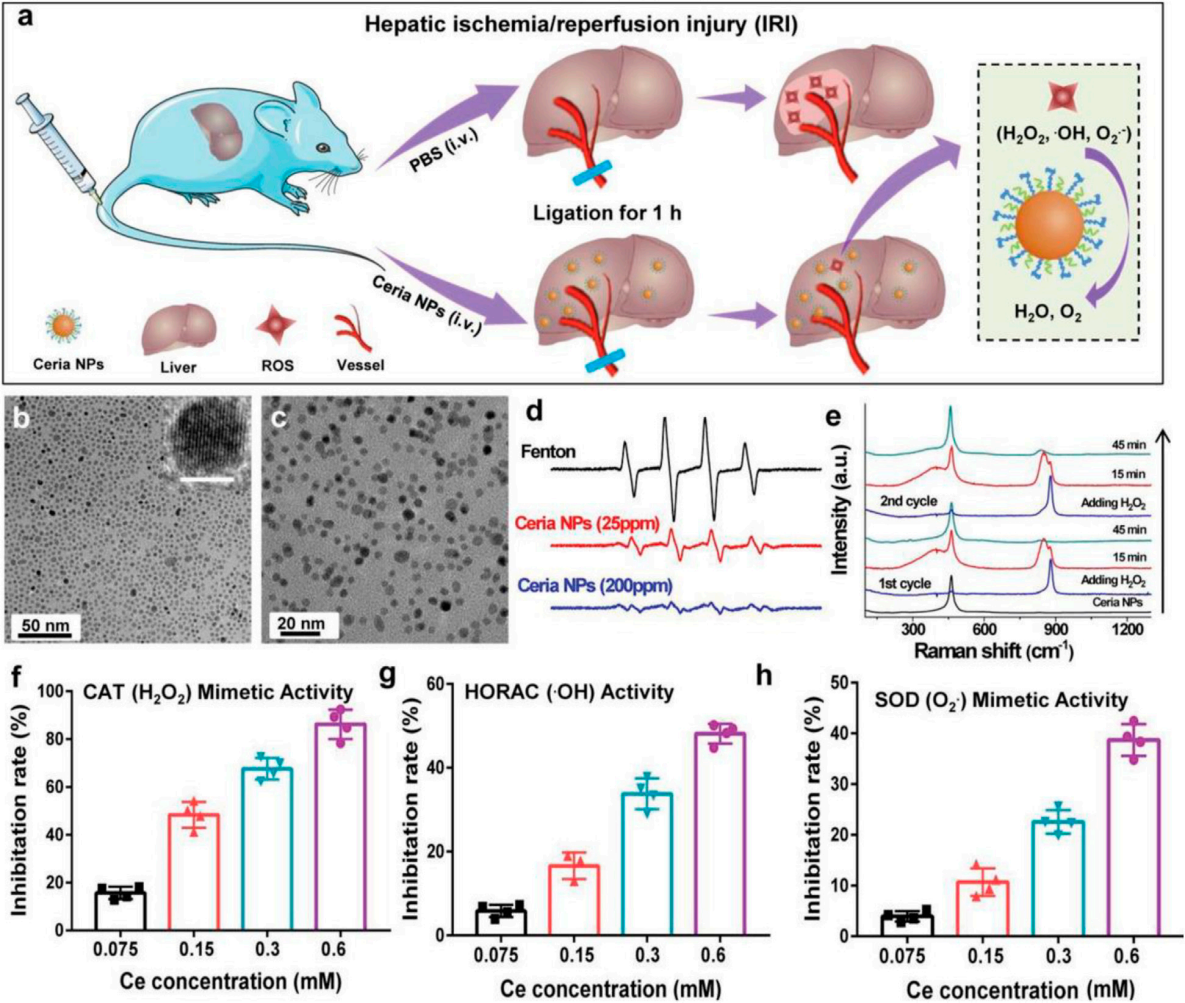
Schematic diagram of ceria in the treatment of IRI and the antioxidant activity of ceria. **(A)** Schematic illustration of ceria for preventing IRI. **(B,C)** TEM images of ceria NPs. **(D)** ESR spectra of the different groups. **(E)** Raman spectra of ceria NPs reacting with H_2_O_2_. **(F–H)** Detection of ROS scavenging ability of ceria NPs. Adapted with permission from ref.60. Copyright (2019) John Wiley and Sons.

Hepatic ischemia-reperfusion injury (IRI) is one of the crucial causes of liver injury during liver transplantation, resection, and hypovolemic shock. Excessive production of reactive oxygen species is an essential factor leading to liver ischemia-reperfusion injury (IRI) (Eltzschig and Eckle, 2011; Zhai et al., 2013). Antioxidant treatment to improve the oxidative stress state of the injured liver is an effective therapeutic measure to improve IRI. The mononuclear phagocytic cell system (MPS) can affect the delivery of nanomaterials to the desired disease area (Bourquin et al., 2018; Feliu et al., 2016) (Figure 4). The direct prevention of liver ischemia-reperfusion injury (IRI) through nano-antioxidants with priority for liver uptake is an important research direction for applying nanomaterials in the treatment of liver IRI. Combining the redox activity characteristics of cerium oxide nanoparticles and the advantages of targeted enrichment in disease regions, Dalong Ni et al. selected representative nano-antioxidant cerium oxide nanoparticles (Ni et al., 2019). The establishment of the mouse liver IRI model specifically discussed the specific mechanism of cerium oxide NPs to prevent IRI and introduced the method of using the black box effect of nanomaterials to treat the live animal liver IRI. Positron emission tomography (PET) imaging real-time noninvasive assessment of cerium oxide NP biological distribution, using radionuclide 89 ^zr^ to mark ceria NPs, proves that polyethylene glycol (PEG) is changed to make these cerium oxides on the surface of the NPs with good blood circulation. Quantitative PET image analysis shows that NPs were cleared from the liver of mice from 1 to 21 days. These characteristics show that cerium oxide has the character of explicit according to the long-term accumulation in the liver to support the applications of cerium oxide in the body against hepatic IRI. The protective effect of cerium dioxide NPs on IRI in the mouse liver IRI model was compared. Aspartate aminotransferase (AST) and alanine aminotransferase (ALT) results showed liver injury in untreated IRI mice and effective prevention of IRI by cerium oxide NPs in the treated group. HE staining of the liver tissue showed liver sections of IRI mice in the PBS group, showing large areas of severe damage and significant lipolysis, necrosis, and bleeding of liver cells. The liver tissues of the IRI group treated with cerium oxide NPs were only slightly damaged, which indicated that cerium oxide NPs had a protective effect on IRI and proved the biocompatibility of cerium oxide NPs *in vivo*. The team’s research will also provide references for the potential applications of nano-anticoagulants in preventing the liver IRI (Ni et al., 2019).

**FIGURE 5:**
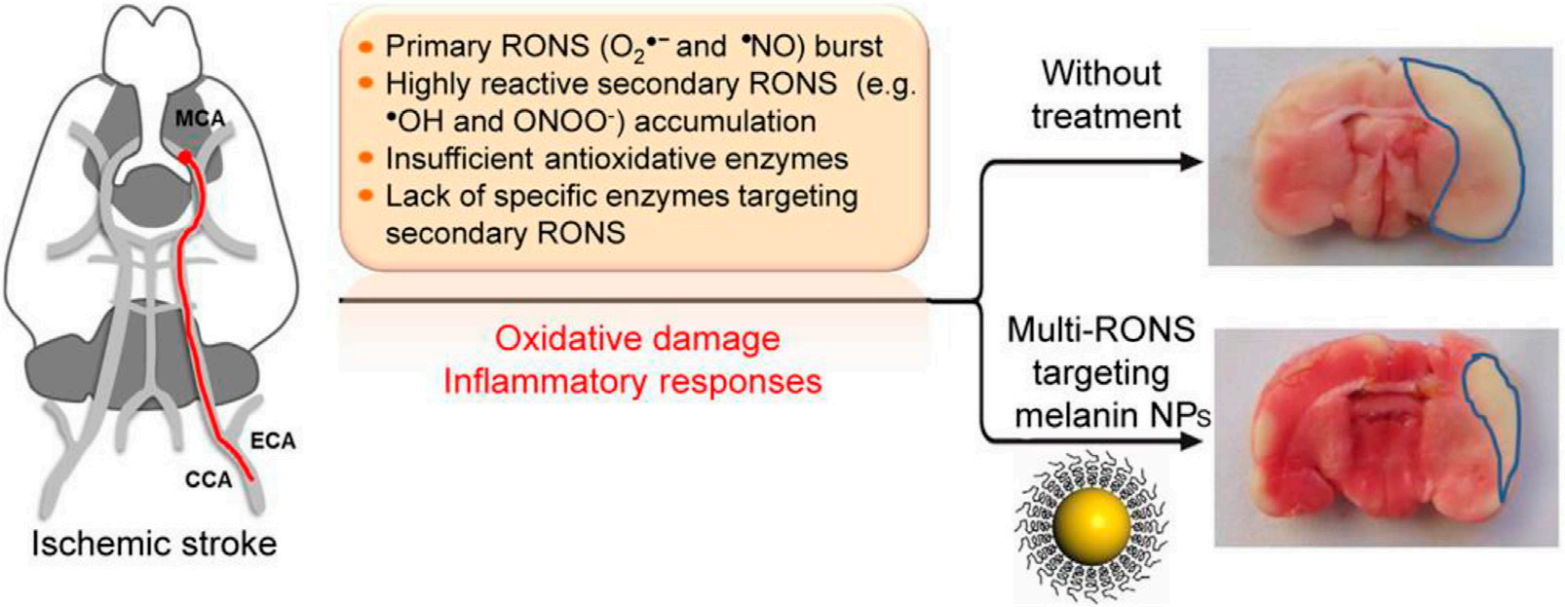
Mechanism and effect of MeNPs in the treatment of ischemic stroke. Adapted with permission from ref.64. Copyright (2017) American Chemical Society.

Acute ischemic cerebrovascular syndrome, also known as ischemic stroke, is a severe cerebrovascular disease that can lead to loss of brain function, disability, and even death. Oxidative damage is one of the important mechanisms leading to ischemic brain injury (Ngowi et al., 2021). During ischemia, the accumulation of superoxide anions, hydroxyl radicals, and hydrogen peroxide induces oxidative damage and leads to cell apoptosis (Barnham et al., 2004; Nathan and Cunningham-Bussel, 2013). Yanlan Liu et al. studied the antioxidant effects of melanin nanoparticles (MeNPs) (Liu et al., 2017) (Figure 5). PEG-coated MeNPs were used to synthesize PEG-MeNPs with antioxidant and anti-inflammatory effects to conduct *in vitro* experiments and establish a rat model of ischemic stroke to study its antioxidant and anti-inflammatory effects *in vitro* and *in vivo*. Liu et al. first identified the antioxidant activity of MeNPs through comprehensive *in vivo* also *in vitro* experiments, demonstrating the feasibility of using these NPs as antioxidants *in vivo*. Liu et al. prepared the highly biocompatible cerium dioxide nanoparticles by coating them with PEG from nanoparticles dispersed in chloroform. TEM and DEPMPO characterized MeNPs and PEG-MeNPs, and SOD-like enzyme activity was found. X-ray photoelectron spectroscopy (XPS) found only a slight increase in the relative oxygen content of PEG-MeNPs. EPR analysis further confirmed the solid free radical signal of PEG-MeNPs. At the same time, PEG-MeNPs could maintain a high catalytic activity even after being stored at 4° for one year. These results indicate that PEG-MeNPs have good antioxidant stability. *In vitro*, neuro 2A cells were cultured using cobalt chloride (CoCl_2_) to evaluate PEG-maps’ neuroprotective effect on ischemia directly. Cobalt chloride (CoCl_2_) is a common *in vitro* model used to investigate the mechanism of antioxidation therapy for the ischemia-related neuronal diseases. CoCl_2_ significantly increased the ROS levels in neuro 2A, followed by cell death due to oxidative stress, while PEG-MeNPs significantly reduced the intracellular ROS levels, demonstrating the antioxidant capacity of PEG-MeNPs in cells. *In vivo*, PEG-MeNPs were injected into the lateral ventricles of experimental rats to evaluate their efficacy in the ischemic brain of experimental rats. The cerebral tissue sections of rats and TTC staining showed that the area of ischemic cerebral infarction of rats pretreated with PEG-MeNPs was about 14%.

Nevertheless, that of the control group was about 32%. The area of cerebral infarction of rats in the PEG-MeNPs-pretreated group was significantly lower than that in the control group. Accordingly, the generation of O_2_ was also effectively inhibited, proving the possibility of treating ischemic brain injury with PEG-MeNPs. Through the tail vein injection of PEG–MeNPs, security in the body was assessed, and it was found that PEG-MeNPs have good blood compatibility, and do not change in serum alanine aminotransferase (ALT), aspartate aminotransferase (AST), and alkaline phosphatase (ALKP), blood in urine nitrogen (BUN), total protein (TP), or the level of albumin (propagated) in different organs of the histological analysis showed no NP morphological changes after the treatment or signs of inflammation, and proved that cerium oxide nanoparticles can be used as a safe and effective antioxidant for the feasibility of the application in the clinical treatment.

### 1.3 Iron-Based NPs

Prussian blue (PB), also known as ferric ferrocyanide, is the only antidote for clinical thallium poisoning, approved by the food and drug administration (FDA). In recent years, different types and functional nanomaterials based on PB have been widely used in inflammatory therapy, tumor therapy, molecular imaging, and other biomedical safety fields (Patra, 2016). PB can simulate various enzyme activities, including SOD, CAT, and POD. For example, Zhang W et al. found that Prussian blue nanoparticles (PBNs) can catalyze H_2_O_2_ with the artificial POD activity (Zhang et al., 2014). Further study by the team found that PBNP does not produce ROS such as OH through Fenton reaction, but has the POD activity of inhibiting the generation of OH, as well as a variety of biological enzyme activities such as CAT and SOD, which can effectively remove a variety of ROS ordinarily, and is widely used in inhibiting cell damage caused by ROS.

**FIGURE 6:**
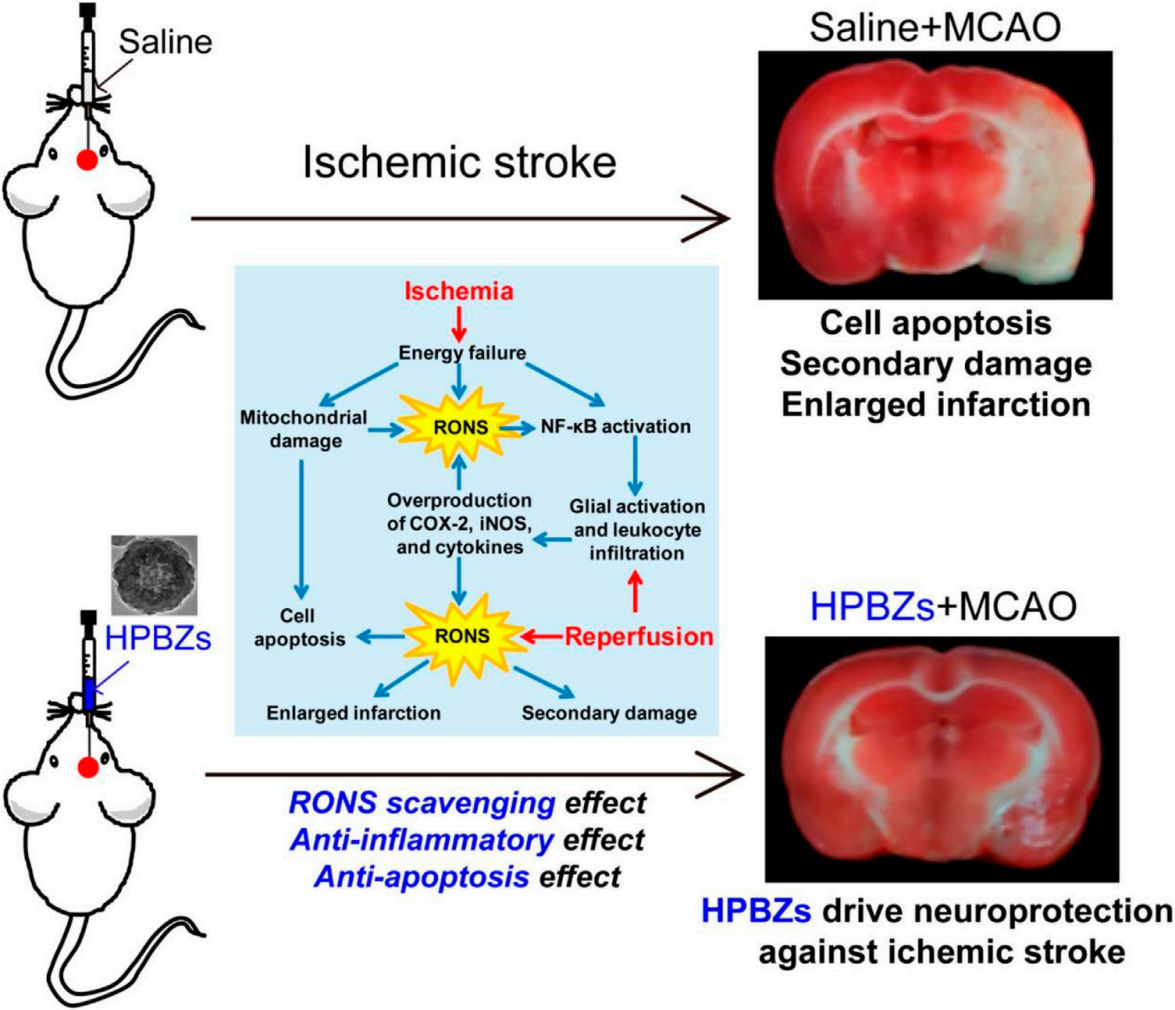
Schematic illustration of HPBZs preventing ischemic stroke-induced neurological damage. Adapted with permission from ref.72. Copyright (2019) American Chemical Society.

The mechanism of ROS scavenging by PB nanozyme particles shows that the surface volume of the nanozyme is a crucial factor in determining ROS scavenging efficiency. Consequently, hollow PB nanozyme is considered to have good ROS scavenging efficiency (Li and Shi, 2014; Wang et al., 2016b). The crystallinity of the hollow PB nanozyme particles synthesized by the microemulsion method is poor. Accordingly, they need to be synthesized in multiple steps at high temperatures. More importantly, it is not easy to achieve mass production (McHale et al., 2010; Roy et al., 2011). Under this background, Kai Zhang et al. based on ischemic stroke disease pathogenesis with ROS, proved that the relationship between the development of the preparation of low cost also can be large-scale production of the artificial hollow nanozyme (HPBZs). The study of a new type of neural protection nano preparation provides the proof of the concept. Moreover, it may be helpful in the treatment of other ischemic stroke diseases related to ROS (Zhang et al., 2019) (Figure 6). The extensive characterization experiments by transmission electron microscopy showed that HPBZs had good crystallization. Accordingly, the changes in the hydrodynamic diameter of HPBZs at different time points proved the stability in the physiological environment and the potential for further application *in vivo* and *in vitro*. HPBZs can catalyze H_2_O_2_ to oxidize ABTS and TMB to nontoxic substances, which proves that HPBZs have an activity similar to peroxides. Choosing the TiO_2_/UV system, HPBZs reduced the characteristic signal strength of BMPO/OH, indicating that HPBZs have good OH scavenging ability. To further demonstrate the antioxidant and anti-inflammatory activities of HPBZs, Kai Zhang et al. conducted cell and animal experiments *in vitro*. Using cobalt chloride (CoCl_2_) to culture the SHSY-5Y cell line and construct an *in vitro* hypoxic model, it was found that HPBZs significantly promoted cell survival and also inhibited the production of reactive oxygen species. These results indicated that HPBZs could promote cell protection through antioxidant activity. Considering HPBZ *in vitro* antioxidant and anti-inflammatory activity, resistance to apoptosis, HPBZs’ team further studied ischemic stroke in rats induced by the middle cerebral artery disease-modifying function in the model. Through the PET experiment, it was found that three pretreatment HPBZs lower ischemic injury in rats, cerebral infarction significantly less than the injected saline volume of MCAO rats, prompt HPBZ pretreatment can improve the nerve dysfunction; further comparing three groups of rats brain NO level and antioxidant capacity, HPBZ pretreatment can effectively suppress the RNS induced by MCAO generates, The anti-ROS activity of the brain tissue was significantly increased. Consequently, these results demonstrated the antioxidant properties of HPBZs in the treatment of ischemic stroke *in vivo*. In summary, Kai Zhang et al. constructed monodisperse HPBZs with good crystallization using a simple, formless synthesis strategy and applied it to treating ischemic stroke in rats, guiding the large-scale application of PB nanoparticles.

**FIGURE 7:**
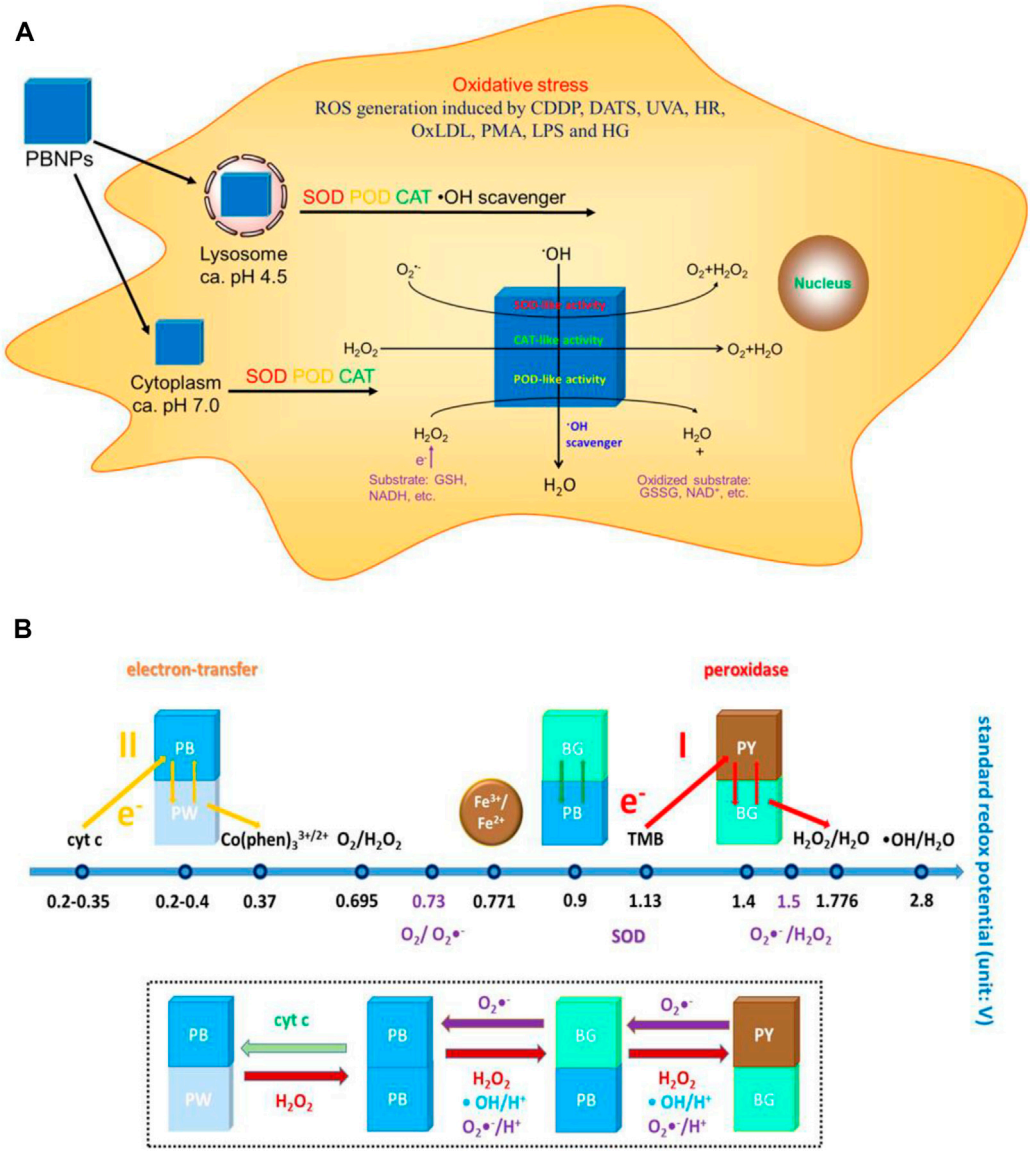
**(A)** Schematic diagram of the mechanism of action of PBNPs in the cells. **(B)** Schematic diagram of the activity of PBNPs with different redox potentials of different compounds in different reaction systems. Adapted with permission from ref.27. Copyright (2016) American Chemical Society.

As mentioned previously, targeting intestinal inflammation and clearing ROS play key roles in IBD treatment. CAT, GPX, SOD, POD, and other antioxidant enzymes are unique proteins with catalytic activity and high selectivity, and they all require the participation of the iron-containing auxiliary groups in the construction to exert excellent catalytic activity (Wu et al., 2019). Therefore, iron-based nanoenzymes have become the focus of research in recent years. On this basis, Zhang W et al. (Zhang et al., 2016) (Figure 7) designed a Prussian blue nanoparticle (PBNP) with a diameter of about 50 nm, which has activities of SOD, POD, CAT, and other enzymes, which can effectively remove ROS, and can effectively control the inflammation *in vivo* of lipopolysaccharide (LPS)-induced inflammatory mice by injection through the tail vein. PBNPs can target the aggregation of DSS-induced mouse colon inflammatory tissues, exert the activity of artificial nanozyme, effectively remove the excessive ROS produced in inflammatory tissues, block ROS-related inflammatory response and oxidative stress response, and inhibit the colon’s progression, which improves the level of intestinal inflammation.

**FIGURE 8:**
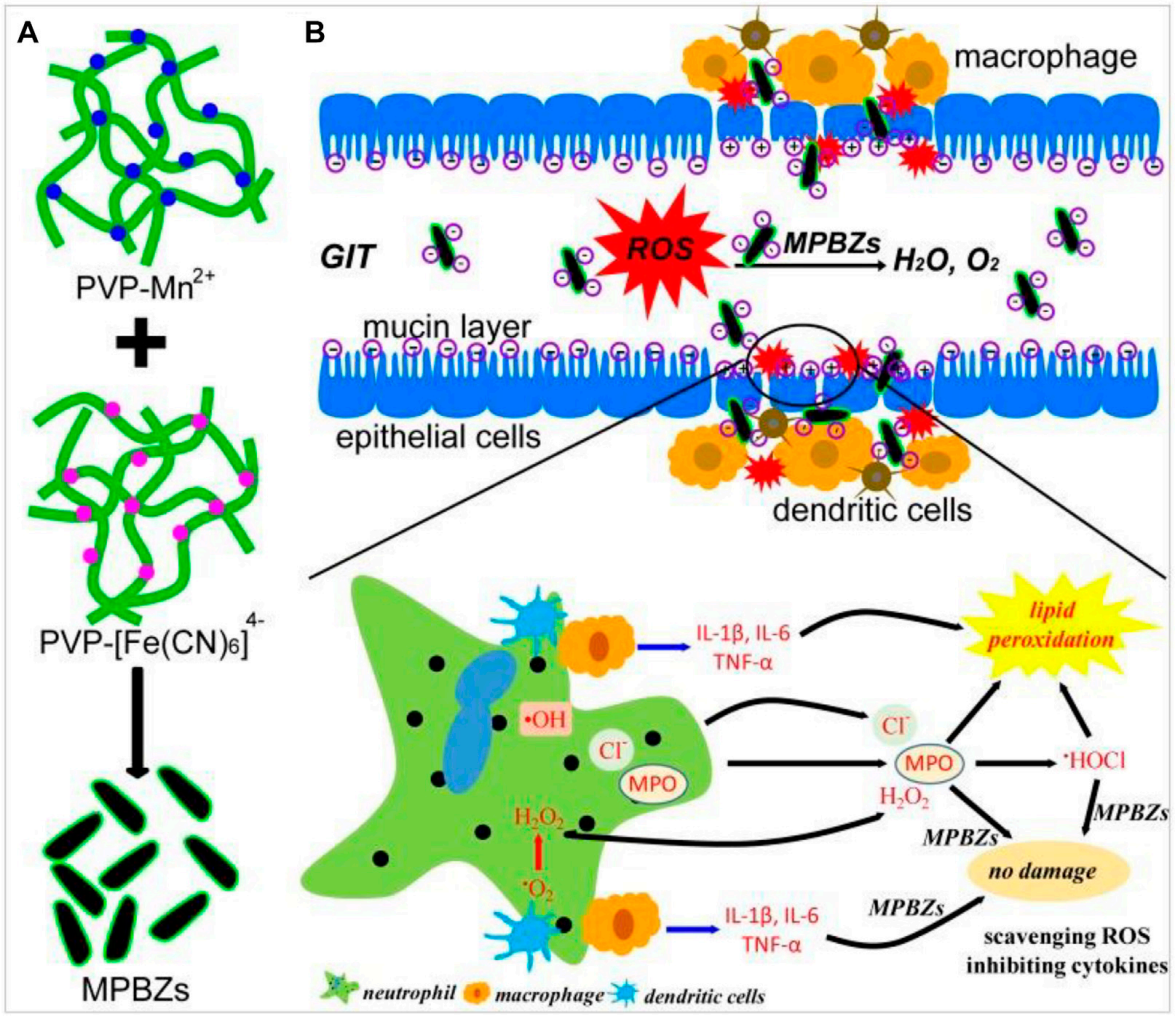
**(A)** Schematic diagram of the synthetic production of MPBZs. **(B)** Schematic diagram of the mechanism of action of MPBZs *in vivo*. Adapted with permission from ref.56. Copyright (2019) Theranostics.

Later, Zhao Jiulong et al. (Zhao et al., 2019) (Figure 8) developed a new and effective strategy of nanoenzyme-catalyzed nanotherapy for IBD. Based on the activity of PBNPs, a novel manganese-Prussian blue nanoenzyme (MPBZs) was synthesized by using PBNPs as the basic framework and introducing Mn^2+^ with solid productivity. With the nanoscale size and good physiological stability, MPBZs can reach the inflammatory site of the colon smoothly through the robust acidic environment in the stomach and alkaline environment in the intestine, and specifically accumulate in the inflammatory site of the colon by using the EPR effect and charge effect of the intestinal epithelium. Due to the introduction of Mn^2+^ in MPBZs, the reducibility of MPBZs was more substantial than that of PBNPs, and the ROS scavenging ability was improved. Therefore, after the oral administration of MPBZs in DSS-induced acute IBD mice, the drugs can target intestinal inflammatory tissues, effectively remove ROS, and improve intestinal inflammation. At the same time, MPBZs can effectively reduce the expression levels of MPO, MDA, IL-1β, IL-6, IFN-γ, and TNF-α in the inflammatory colon tissue and effectively inhibit the progression of inflammation. Moreover, Zhao Jiulong synthetic MPBZs have many kinds of enzymes such as SOD and CAT activity, which can effectively eliminate ROS, are a high-quality artificial enzyme, inhibit oxidative stress in the process of inflammation reaction, and affect the oxidation of inflammatory-related signaling pathways and related critical molecular expression. Signaling pathway to reduce inflammation, especially for the ROS-related diseases, IBD provides a new thought of treatment, which has broad application prospects.

### 1.4 Other Nanomaterials

#### 1.4.1 Platinum-Based NPs

Pt-NPs have activities similar to mitochondrial electron transport complexes and can be used as inhibitors of SOD and catalase. Watanabe et al. studied the antioxidant effects of PAA-protected platinum nanoparticles (PAA-Pt) (Watanabe et al., 2009). In *in vitro* evaluation, PAA-Pt cleared AOO generated by AAPH thermal decomposition in a dose-dependent manner. The concentration of NPs at 50% of any event was recovered (IC_50_) 584 M, while the control showed no antioxidant activity. PAA-Pt is at least six times more active than other metal nanoparticles. The inhibition of PAA-Pt on linoleic acid peroxidation induced by AOO was further evaluated by determining the oxygen consumption and phenobarbital acid reactive substance (tar). Oxygen consumption decreased significantly after adding NPs, but there was no significant difference between oxygen consumption and the control group. The authors suggest that the peroxide inhibition mechanism of PAA-Pt particles may mainly be the elimination of toilets, which inhibits the peroxidation of linoleic acid. The thiobarbituric acid (TBA) test was designed to assess the production of free malondialdehyde (MDA) during lipid peroxidation. Accordingly, the results showed that PAA-Pt reduced the production of lipid peroxides by inhibiting the proliferation of the nitrite peroxidation chain induced by AOO. *In vivo*, Pt-NPs also act as a scavenger for RON. Katsumi et al. demonstrated for the first time in a mouse model that these NPs could protect against the hepatic ischemia-reperfusion injury (Katsumi et al., 2014). In their study, two Pt-NPs of different sizes were injected intravenously into mice, causing liver damage by blocking the portal vein, and consequently then reperfused for six hours. The results showed that both NPs were accumulated in the liver non-parenchymal cells after injection. The smaller the Pt-NPs, the more significant the decrease in alanine aminotransferase (ALT) and aspartic aminotransferase (AST). Small NPs also inhibited the increase in the ratio of oxidized GSH to reduced glutathione in the ischemic liver, effectively reducing the increase in lipid peroxides.

#### 1.4.2 Selenium-Based NPs

As part of the liver’s antioxidant defense system, selenium plays an essential role in antioxidative stress. Many studies have shown that supplementation with selenium can increase enzymes such as GPx, preventing the accumulation of free radicals and thus reducing cell damage (Tapiero et al., 2003; Qin et al., 2015). Therefore, nanomaterials containing selenium have intrinsic antioxidant properties. Zhai et al. used chitosan (CS) of different molecular weights to stabilize the synthesis of SeNPs, and then evaluated the antioxidant capacity of these nanoparticles (Zhai et al., 2017). The results of *in vitro* cell tests showed that the generation of intracellular electrons was inhibited by selenium concentration, also cs-sends; CS(l)-sends were stable by external or oral use, which effectively protected the glutathione peroxidase activity in mice and accordingly prevented the formation of lipofuscin induced by ultraviolet light. Se-doped carbon quantum dots (Se-CQDs) are also scavenger free radicals (Rosenkrans et al., 2020). In a recent study, the radical scavenging ability of SE-CQDs was investigated using the electron spin resonance (ESR) technique. The results showed that after the addition of SE-CQDs, the ESR signal of the DEMPO/OH adder disappeared, which indicated that it had an intense OH scavenging activity. The *in vitro* cell models have shown that SE-CQDs can protect MDA-MB-231 cells from H_2_O_2_-induced oxidative stress. This was achieved by reducing H_2_O_2_-induced cell death consequently increasing cell viability (as determined by CCK8). In addition, quantitative analysis of ROS induction in MDA-MB-231 cells using the RONS fluorescent probe (DCFH-DA) showed that the fluorescence intensity of DCFH-DA significantly decreased after the addition, which confirmed the antioxidant capacity of SE-CQDs.

## 2 Conclusion

Reactive oxygen species (ROS) are metabolites of normal cells in organisms. Normal levels of ROS in cells are very important for maintaining cell signaling and other intracellular functions, so the dysregulation of ROS balance is involved in the pathophysiological processes of many diseases. The ideal antioxidant nanomaterials should be able to remove multiple primary and secondary electrons, maintain antioxidant activity against oxidative damage, be biocompatible, and have controllable properties such as size and modifiable surface. Choosing the proper nanomaterials for each disease is complicated because each disease has its unique process of oxidative stress, not the same, which is the most critical factor affecting the outcome of the disease. The selection of suitable nanomaterials for ROS scavenging should be based on their specific antioxidant capacity and their biological distribution, cyclic half-life, immunological properties, and other *in vivo* pharmacokinetics. With the development of nanometer research, various nanomaterials have been developed. Therefore, it is desirable to explore the antioxidant properties of these nanomaterials through a large number of *in vivo* studies.
